# Near Resonant Raman Optical Activity Study of the Complex Cation Tris(ethylenediamine)cobalt(III) in Water: Experiment and DFT Calculation

**DOI:** 10.1002/chir.70128

**Published:** 2026-08-27

**Authors:** Maria Sara Raju, Nazanin Kordestani, Elen Duverger‐Nédellec, Yann Danten, Patrick Rosa, Elizabeth A. Hillard, Nicolas Daugey

**Affiliations:** ^1^ ISM, UMR 5255 CNRS, Bordeaux‐INP, Univ. Bordeaux Talence France; ^2^ ICMCB, UMR 5026 CNRS, Bordeaux‐INP, Univ. Bordeaux Pessac France

**Keywords:** chirality, cobalt(III), conformational analysis, ECD, Raman, Raman optical activity

## Abstract

Chiral metal complexes are important in catalysis and medicinal chemistry, where understanding their structural and optical properties is crucial. This study presents a combined experimental and theoretical Raman optical activity (ROA) investigation of the chiral tris(ethylenediamine)cobalt(III) complex, Δ‐ and Λ‐[Co(en)_3_]^3+^, in aqueous solution. ROA spectra were recorded using near‐resonance excitation at 532 nm. Under these conditions, a significant ECD–Raman contribution from the solution was observed and quantitatively corrected, enabling extraction of the intrinsic ROA spectra. Density functional theory (DFT) and time‐dependent DFT (TD‐DFT) calculations were used to simulate ROA, Raman, UV–visible absorption, and electronic circular dichroism (ECD) spectra. Four representative conformers were analyzed, and their stability and spectral properties were evaluated using both the polarizable continuum model (PCM) and the solvation model based on density (SMD). While both models reproduced key experimental trends, SMD provided a more accurate description of vibrational features, especially NH_2_ scissoring and CH_2_ rocking modes. This work shows that near‐resonant ROA spectra of a cationic transition‐metal complex in water can be accurately reproduced using first‐principles methods without empirical adjustments, highlighting the importance of conformational averaging and solvation effects.

## Graphical abstract

Corrected ROA spectrum of the ECD‐Raman (red curve) from the observed raw signal (black curve) reveals the intrinsic ROA signal of Δ‐[Co(en)_3_]^3+^, enabling an excellent theory‐experiment agreement.

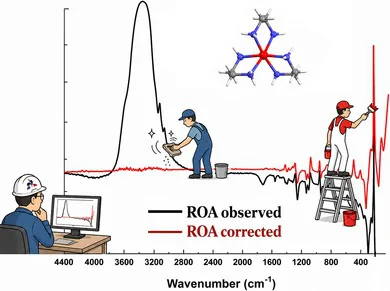

## 1 Introduction

Metal complexes play a central role in a wide range of scientific and technological applications, spanning catalysis [1], materials science, and therapeutic interventions [2]. A notable example is vitamin B_12_, which exists as a cobalt complex [3], whereas coordination compounds of transition metals such as Cu(II), Fe(III), and Mn(II) have been extensively explored for their relevance in medicinal chemistry [4]. Owing to their multiple oxidation states, coordination numbers, and tunable physicochemical properties, transition metal complexes exhibit remarkable versatility, giving rise to a large body of research towards the design of new ligands and complexes with tailored physical, chemical, and biological properties [5].

The introduction of chirality into metal complexes further expands this versatility, opening new avenues for both fundamental and applied research. Rationally designed chiral metal complexes [6] have been shown to exhibit a range of emergent phenomena, including second harmonic generation [7], ferroelectricity [8], magneto‐chiral effects [9], and multifunctionality [10], thereby giving rise to materials with potential applications in optics, electronics, and sensing. In addition, chiral complexes also serve as powerful sources of asymmetry in catalytic systems, where they play a critical role in controlling stereoselectivity in asymmetric organic synthesis [11].

Given this breadth of application, experimental techniques capable of providing detailed insight into the structure and properties of chiral metal complexes are of paramount importance. Among a variety of analytical methods, chiroptical spectroscopies have emerged as particularly powerful tools for probing chirality in coordination compounds. Electronic circular dichroism (ECD), in particular, is widely used to investigate electronic structure and stereochemical features by measuring the differential absorption of left‐ and right‐circularly polarized light (LCPL and RCPL, respectively), thereby providing direct information on molecular chirality and electronic transitions [12].

Beyond electronic chiroptical methods, vibrational otical activity (VOA) provides access to chiral information encoded in the molecular vibrational manifold. VOA encompasses two complementary techniques: vibrational circular dichroism (VCD) and Raman optical activity (ROA) [13]. By directly interrogating vibrational transitions associated with the 3N–6 normal modes, VOA enables a detailed characterization of local geometry, conformational preferences, and absolute configuration. Compared with electronic chiroptical techniques such as ECD or optical rotary dispersion, VOA offers substantially richer structural information owing to the much larger number of accessible vibrational transitions [13]. While VCD has been more widely employed, ROA presents two distinct advantages. The first is access to low‐frequency vibrational modes down to approximately 100 cm^−1^, in contrast to the typical lower limit of ~600 cm^−1^ in VCD. This extended spectral window is particularly valuable for coordination compounds, as it allows direct probing of metal–ligand and coupled vibrational modes. Secondly, ROA measurements can be readily performed in aqueous solution, water being an ideal solvent for ROA spectroscopy, whereas VCD experiments generally require the use of deuterated solvents.

In practice, ROA measures the differential inelastic scattering of RCPL and LCPL by a chiral medium. First predicted theoretically in 1971 [14] and demonstrated experimentally in 1973 [15], ROA has since developed into a powerful tool for detailed structural characterization, with applications ranging from simple organic molecules to complex biomolecular systems such as proteins as well as in virus detection [16] and pathological diagnostics [17]. Despite its potential, the ROA signal contributes to only a tiny fraction (10^−3^–10^−4^) of the total Raman scattering, posing significant experimental challenges for its measurement.

When the excitation wavelength approaches an electronic absorption band (near‐resonance) or coincides with it (resonance), scattering intensities are enhanced, leading to resonance Raman and resonance ROA effects [18, 19]. While resonance conditions may substantially amplify both Raman and ROA signals, electronic absorption also introduces parasitic effects that complicate spectral acquisition and interpretation. These include fluorescence, photodecomposition, reabsorption of Raman‐scattered light, circularly polarized luminescence, and the recently identified ECD–Raman effect [20], which arises from interference between the ECD of a chiral solute and the circularly polarized Raman scattering of the solvent. Briefly, any ECD arising from a chiral solution results in the differential absorption of the right and left circular polarization components of an unpolarized excitation. In this condition, the transmitted excitation is slightly circularly polarized and so generates a residual circularly polarized Raman spectrum of the solvent, which is detected in the raw ROA spectrum. Accurate correction of these contributions is therefore essential to extract reliable ROA spectra under near‐resonant or resonant conditions.

Transition metal complexes are particularly susceptible to such effects, as charge‐transfer or d–d transitions frequently occur in the visible region, placing many systems in near‐resonant or resonant regimes. Theoretical simulations of ROA spectra under near‐resonant or resonant conditions remain challenging, often requiring the inclusion of damping effects, excitation‐frequency shifts, or explicit vibronic coupling treatments [21, 22]. To date, experimental and theoretical ROA investigations have been largely focused on neutral complexes, including systems based on Zr [23], Cu [19, 20, 24, 25], Ni [20], Eu [26, 27], Mn [24], Fe [28], and V [29] with more recent studies focusing on Co complexes [21, 22, 25, 30]. Only one cationic complex, based on Rh(III) [31, 32], studied under far‐from‐resonance conditions, has been experimentally and theoretically reported.

Tris‐chelated Co(III) complexes represent prototypical systems for investigating chiroptical phenomena. Among the first coordination compounds to be resolved into enantiomers [33], these complexes combine structural simplicity with well‐defined stereochemistry, making them particularly well suited for probing structure–property relationships. In particular, cationic tris‐(1,2‐ethylenediamine) complexes, [M(en)_3_]^2+/3+^, represent some of the simplest examples of chiral helicoidal coordination architectures and have been extensively studied with respect to optical rotatory strength [34], ECD [35] and its prediction [36], and determination of absolute configuration via X‐ray diffraction [37], and VCD [38].

In contrast, experimental investigations of ROA for this family of complexes remain scarce. While an experimental and theoretical ROA study of the Rh(III) analogue, [Rh(en)_3_]^3+^, in aqueous solution has been reported [31, 32], the Co(III) complex, [Co(en)_3_]^3+^, has so far been examined only theoretically [39]. Notably, at the commonly employed excitation wavelength of 532 nm in ROA spectroscopy, cobalt(III) tris‐chelated complexes exhibit significant electronic absorption, placing them in a near‐resonant or resonant regime and making them attractive candidates for resonance‐enhanced ROA studies.

**SCHEME 1:**
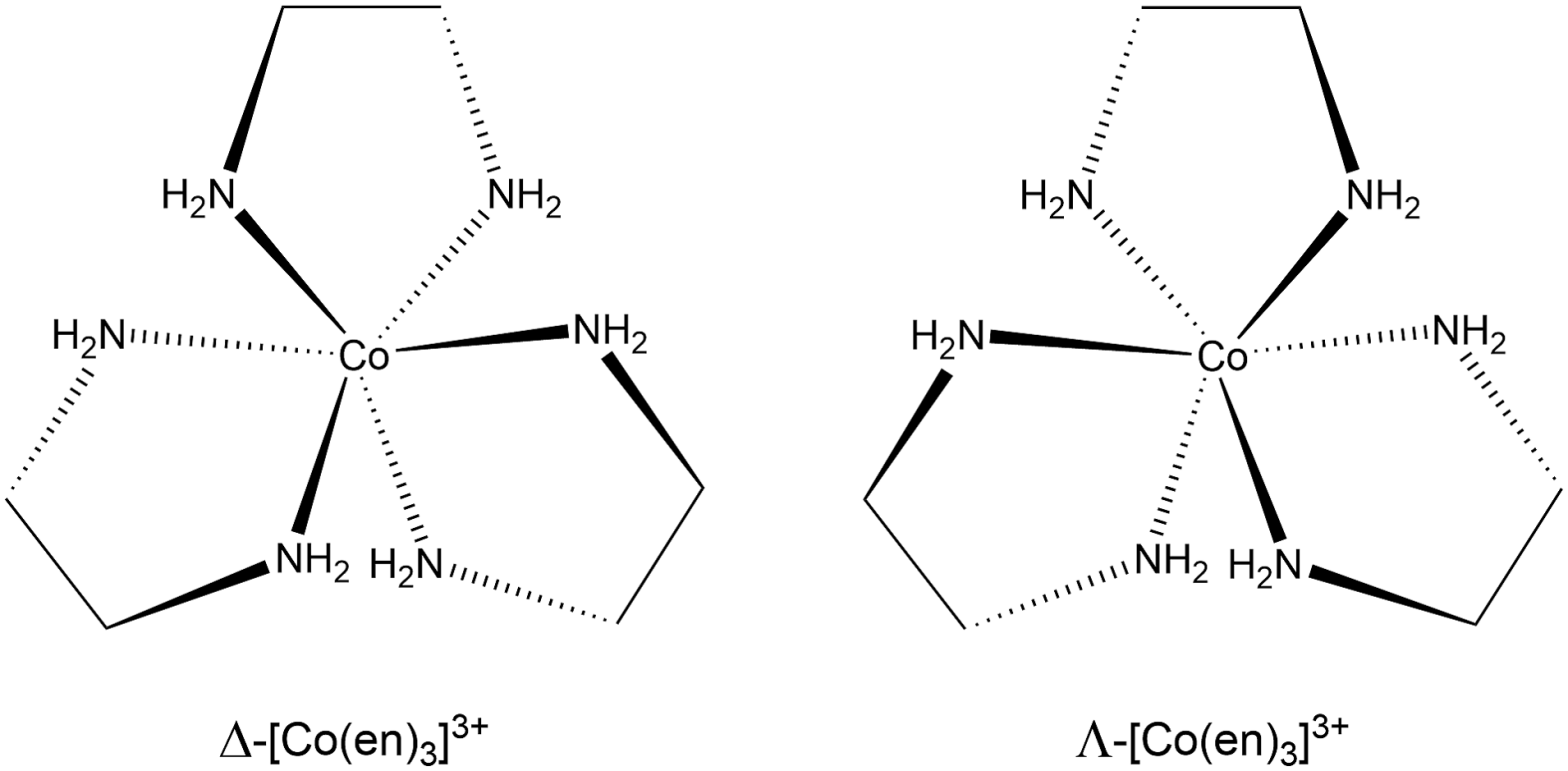
∆‐ and Λ‐[Co(en)_3_]^3+^ enantiomers.

In this work, we report the first experimental ROA spectra in aqueous solution of both enantiomers of a cobalt(III) complex cation (Scheme 1), crystallizing as [Co(en)_3_]^3+^(tartrate)Cl·5H_2_O. We combine UV–visible absorption, ECD, Raman, ROA, and ECD–Raman measurements to obtain a comprehensive chiroptical characterization of the system. The intrinsic ROA spectra of the complex are extracted from the raw experimental data through quantitative correction of the ECD–Raman contribution arising from the solution. These experimental results are interpreted using complementary density functional theory (DFT) and time‐dependent DFT (TD‐DFT) calculations, explicitly accounting for conformational averaging and solvation effects. The present study provides new insight into near‐resonant ROA in chiral transition metal complexes and establishes a robust framework for the combined experimental and theoretical investigation of such systems in solution.

## 2 Materials and Methods

### 2.1 Synthesis

All reagents were obtained from commercial suppliers and used as received. All procedures were carried out under ambient atmospheric conditions. Synthesis of Δ/Λ‐[Co(en)_3_][D(−)/L(+)‐tartrate]Cl·5H_2_O was carried out according to the procedure described by Broomhead et al. [40] with some modifications.

#### 2.1.1 [Λ‐Co(en)_3_][L(+)‐tartrate]Cl·5H_2_O

First, L(+)‐tartaric acid (2.4 mmol, 0.36 g) was added to an aqueous solution of [Co(en)_3_]Cl_3_·3H_2_O (2.0 mmol, 0.67 g). The mixture was stirred at room temperature for 5 min, after which NaOH (5.0 mmol, 0.20 g) was added. The reaction mixture was then gently heated without boiling and stirred for several minutes until all solids had dissolved. Upon cooling to room temperature, brown crystals of the desired complex formed. The crystals were isolated by filtration, washed with cold water, and further purified by recrystallization via slow evaporation of water. [Δ‐Co(en)_3_][D(−)‐tartrate]Cl·5H_2_O was synthesized following the same procedure used for [Λ‐Co(en)_3_][L(+)‐tartrate]Cl·5H_2_O, except that D(−)‐tartaric acid was used in place of L(+)‐tartaric acid.

### 2.2 Physical Methods

#### 2.2.1 Electronic Spectroscopy

UV–visible absorption and ECD measurements were performed on aqueous solutions with a concentration of 56 mM. This high concentration was used to simulate the conditions used for the Raman/ROA measurements. UV–visible absorption spectra were recorded between 250 and 800 nm using a Shimadzu UV‐3600 spectrophotometer and a 2‐mm path‐length quartz cell. ECD spectra were acquired using a JASCO J‐1500 spectropolarimeter equipped with polarimetry cells of 2‐mm path‐length, covering the spectral range from 200 to 800 nm. Data were digitized with a step of 0.5 nm, an integration time of 0.5 s, a scanning speed of 200 nm·min^−1^, and a bandwidth of 2.0 nm. For each enantiomer, six individual scans were recorded and averaged to obtain the final spectra.

#### 2.2.2 Raman and ROA Measurements

Aqueous solutions were prepared at concentrations of 22.9 mg·mL^−1^ for Δ‐[Co(en)_3_](D(−)‐tartrate)Cl·5H_2_O and 19.9 mg·mL^−1^ for Λ‐[Co(en)_3_](L(+)‐tartrate)Cl·5H_2_O and transferred into fused‐silica microcells. Raman and ROA spectra were recorded at ambient temperature using a ZEBR ROA spectrometer [41] equipped with a Cobolt Samba DPSS laser operating at an excitation wavelength of 532 nm. Measurements were performed in backscattering geometry using several polarization modulation schemes, including scattered circular polarization (SCP), incident circular polarization (ICP), dual circular polarization I (DCP_I_), and dual circular polarization II (DCP_II_) [13].

For each sample, Raman and ROA spectra were collected by alternating 32 scans with an illumination time of 2 s in SCP mode, followed by 32 scans in DCP_I_ and DCP_II_ mode. This acquisition protocol enables the evaluation of the ECD–Raman contribution to the measured ROA signal. Spectra were accumulated over the spectral range of 90–4400 cm^−1^ with a spectral resolution of half‐width at half‐maximum (HWHM) of 7 cm^−1^. Measurements were carried out using a laser power of 100 mW during an acquisition time of 39 h for the Δ enantiomer and 130 mW for 24 h for the Λ enantiomer. All spectra were corrected for instrumental response and normalized with respect to laser power and acquisition time. Raman spectra are presented as the sum of intensities (*I*_R_ + *I*_L_), whereas ROA spectra are expressed as intensity differences (*I*_R_ − *I*_L_), where *I*_R_ and *I*_L_ correspond to Raman intensities measured with RCPL and LCPL, respectively.

#### 2.2.3 ECD–Raman Contribution and ROA Correction

At the excitation wavelength of 532 nm, the [Co(en)_3_]^3+^ chromophore exhibits significant electronic absorption, placing the system in the near‐resonance regime. Under these conditions, the raw SCP ROA spectra contain a substantial ECD–Raman contribution arising from the interference between the ECD of the chiral solute and the circularly polarized Raman scattering of the solvent [20]. The experimentally observed ROA signal can therefore be expressed as the sum of the intrinsic ROA contribution of the solute and the ECD–Raman contribution arising from the solution [20, 22, 25, 30].

(1) $${\left(I_{R}^{u}-I_{L}^{u}\right)}_{\mathrm{obs}}={\left(I_{R}^{u}-I_{L}^{u}\right)}_{\text{pureROA}\;\left(\text{solute}\right)}+{\left(I_{R}^{u}-I_{L}^{u}\right)}_{\mathrm{ECD}-\text{Raman}\;\left(\text{solution}\right)}$$

Here, the superscript refers to the polarization state of the incident light, and the subscript refers to the polarization state of the detected light, with *R*, *L*, and *u* denoting right‐circularly polarized, left‐circularly polarized, and unpolarized light, respectively.

The ECD–Raman contribution was evaluated using ECD and circular intensity difference (CID) spectra derived from DCP_I_ and DCP_II_ measurements, following established procedures [30]. This contribution was then subtracted from the raw spectra to extract the intrinsic (“pure”) ROA spectra of the complex. Full details of the correction procedure, including definitions of the CID, DoC, and associated correction factors, are provided in the Supporting Information.

### 2.3 Computational Details

The spectroscopic properties of the cation [Co(en)_3_]^3+^ in aqueous solution were investigated using DFT (Raman and ROA spectra) and TD‐DFT (UV–visible absorption and ECD spectra) calculations as implemented in the Gaussian16 software package [42]. All DFT and TD‐DFT calculations were performed using the hybrid B3LYP exchange–correlation functional [43] in conjunction with the triple‐ζ valence Def2‐TZVP basis set, including cobalt, from the redefined Ahlrichs basis set family [44, 45]. Geometry optimizations of the Δ enantiomer were carried out using tight convergence criteria. Each optimized structure was confirmed as a local minimum on the potential energy surface by verifying the absence of imaginary vibrational frequencies, corresponding to a singlet ground state (S_0_).

*Solvent effects* were taken into account using two distinct implicit solvation models within the integral equation formalism (IEF): (i) the polarizable continuum model (IEFPCM) [46, 47] and (ii) the universal solvation model based on solute electron density (SMD), a refinement of IEFPCM developed by Truhlar and co‐workers [48]. The SMD model, which is generally recommended for estimating Gibbs free energies of solvation and is considered particularly reliable for high‐dielectric solvents, was used to assess solvent stabilization effects in aqueous solution.

#### 2.3.1 Raman and ROA Spectra

Intensities presented in the following figures (see also Figure S1) were computed at 532‐nm excitation using the gauge‐including atomic orbitals (GIAO) approach within the electromagnetic field perturbation framework [49]. However, calculations at other excitation wavelengths were performed and are presented in the Supporting Information: namely, 633, 785, 1064, and 1319 nm (Figure S2) and especially in the range of 465–515 nm (Figures S3–S5) in order to evaluate the location of the resonant ROA spectra (RROA) with our model (using SMD). It is noteworthy that by approaching the absorption maximum, evaluated at about 465 nm (Table S1), the resonant ROA spectra should exhibit both a significant intensity enhancement as well as a mono‐signate signature (instead of bisignate in near and far resonance) [13]. In our work, we shall distinguish the near‐resonant ROA spectra obtained at 532‐nm excitation, situated towards the edge of the absorption band (as shown in the following sections), from the resonant ROA spectra rigorously obtained at the absorption maximum of the sample situated at ~465 nm.

Based on the optimized Δ‐[Co(en)_3_]^3+^ geometries, UV–visible absorption and ECD spectra were subsequently calculated using the TD‐B3LYP linear‐response formalism, considering the lowest 48 singlet electronic excitations. Simulated electronic spectra were generated by applying a Gaussian profile broadening of 0.20 eV to the calculated vertical excitation energies.

#### 2.3.2 Conformational Analysis

**FIGURE 1:**
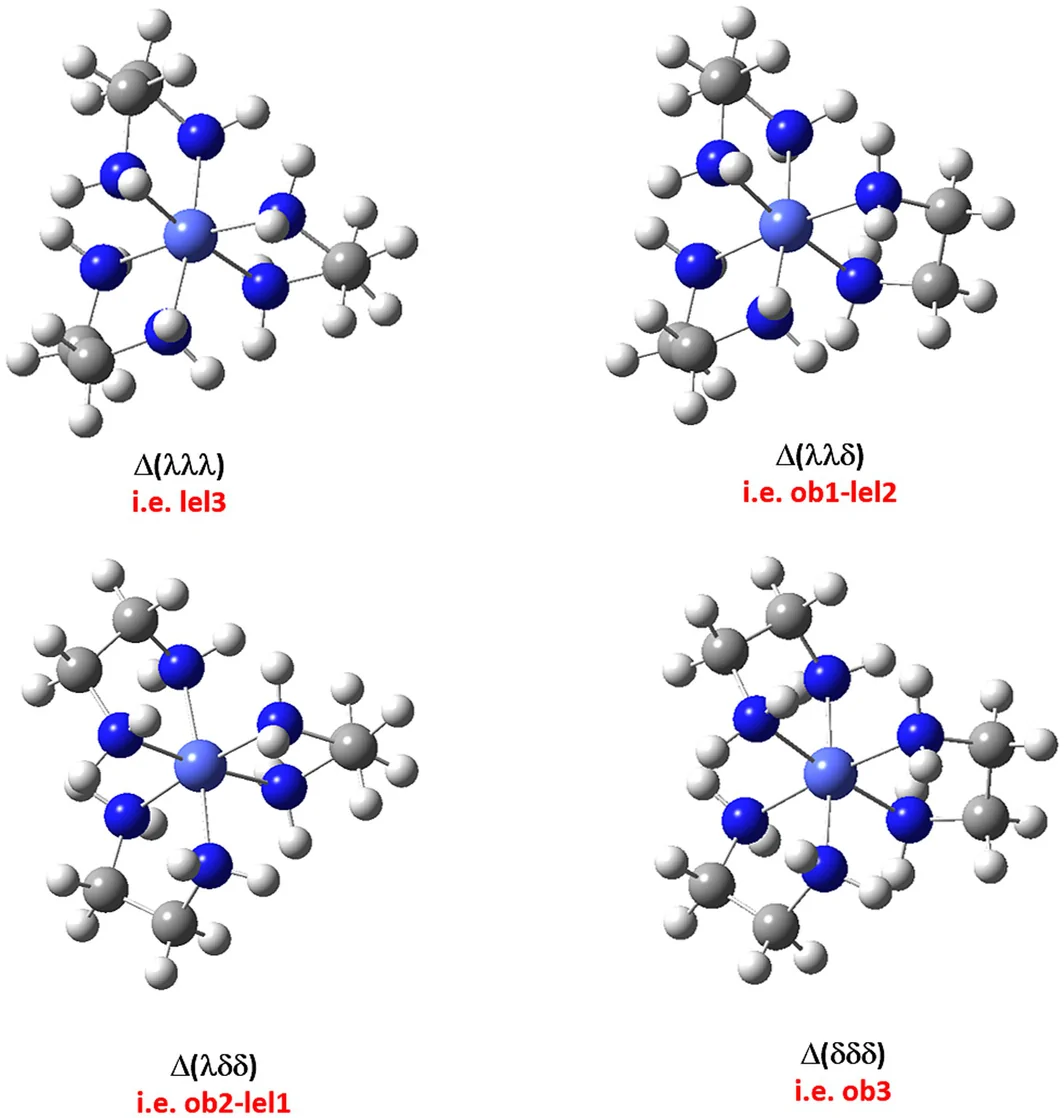
Schematic representation of the four conformers ∆‐[Co(en)_3_]^3+^ from the possible δ and λ conformations of the M‐ligand bonds calculated at the B3LYP/Def2TZVP using both PCM and SMD solvent models for water.

Four representative conformations of Δ‐[Co(en)_3_]^3+^ in aqueous solution were considered. These conformers are denoted Δ(λλλ) (lel_3_), Δ(λλδ) (lel_2_ob_1_), Δ(λδδ) (lel_1_ob_2_), and Δ(δδδ) (ob_3_), following the nomenclature adopted in the literature [31, 38, 50]. Schematic representations of these structures are shown in Figure 1.

Within this nomenclature, each achiral en ligand can adopt two distinct conformations upon chelation to the metal center, designated δ or λ, depending on the sign of the NCCN dihedral angle γ. By convention, γ > 0 corresponds to the δ conformation and γ < 0 to the λ conformation. In tris‐chelated complexes, the δ and λ conformations are not energetically equivalent due to steric and electronic interactions between neighboring chelated ligands.

For the Δ‐[Co(en)_3_]^3+^ enantiomer, the λλλ conformation corresponds to a structure in which the C–C bonds of all three ligands are oriented approximately parallel to the C_3_ symmetry axis, Δ(λλλ) or lel_3_. In contrast, the δδδ conformation corresponds to a structure in which the C–C bonds of the ligands are obliquely oriented relative to the C_3_ axis, Δ(δδδ) or ob_3_. The intermediate λλδ and λδδ conformations represent hybrid structures in which the C–C bonds of the ligands exhibit mixed orientations with respect to the threefold axis and are denoted Δ(λλδ) (lel_2_ob_1_) and Δ(λδδ) (lel_1_ob_2_), respectively.

For the Λ‐[Co(en)_3_]^3+^ enantiomer, the sign of the dihedral angle γ is opposite (γ → −γ), such that the assignments of the δ and λ conformations are interchanged. Consequently, for Λ complexes, the conformations corresponding to lel_3_ and ob_3_ are Λ(δδδ) and Λ(λλλ), respectively, reflecting the reversed orientation of the C–C bonds relative to the C_3_ axis. In the present study, explicit DFT calculations were performed only for the Δ enantiomer. By symmetry considerations, the corresponding properties of the Λ enantiomer can be obtained through a simple δ ↔ λ conformational transformation, and so the enantiomer of Δ(δδδ) (ob_3_) is then Λ(λλλ) (ob_3_).

#### 2.3.3 Thermodynamic Populations

For both Δ and Λ enantiomers, the relative population of each conformer in solution was evaluated using a Maxwell–Boltzmann distribution,

(2) $$p_{i}=\frac{g_{i}e^{-\Delta E_{i}/k_{B}T}}{\sum_{i}g_{i}e^{-{\Delta E}_{i}/k_{B}T}}$$

such that ∑_i_ *p*_i_ = 1.

Here, *p*_i_ is the population of conformer *i*, *g*_*i*_ is its degeneracy, and Δ*E*_*i*_ is its relative Gibbs free energy determined by DFT calculations. The hybrid conformers λδδ and λλδ possess a degeneracy factor of *g*_*i*_ = 3, corresponding to the three possible choices of the ethylenediamine ligand adopting either a λ or δ conformation. As a result, these hybrid forms are entropically favored (by a factor of RTln(3)) relative to the pure λλλ and δδδ conformers.

The calculated structural and thermodynamic parameters of the Δ‐[Co(en)_3_]^3+^ conformers, including electronic energies *E*_0_, zero‐point energy–corrected values *E*_0_ + ZPE, internal energies *U*_298 K_, enthalpies *H*_298 K_, and Gibbs free energies *G*_298 K_, together with the corresponding estimated conformer populations, are reported in Tables S2 and S3 for both implicit solvent models PCM and SMD. Relative conformer stabilities were determined on the basis of their Gibbs free energies at 298 K. Transition States energies for δ‐ (γ > 0) → λ‐ (γ < 0) transformations between conformers were estimated (Figure S6 and Table S4).

## 3 Results and Discussion

### 3.1 Experimental Raman and ROA Spectra

**FIGURE 2:**
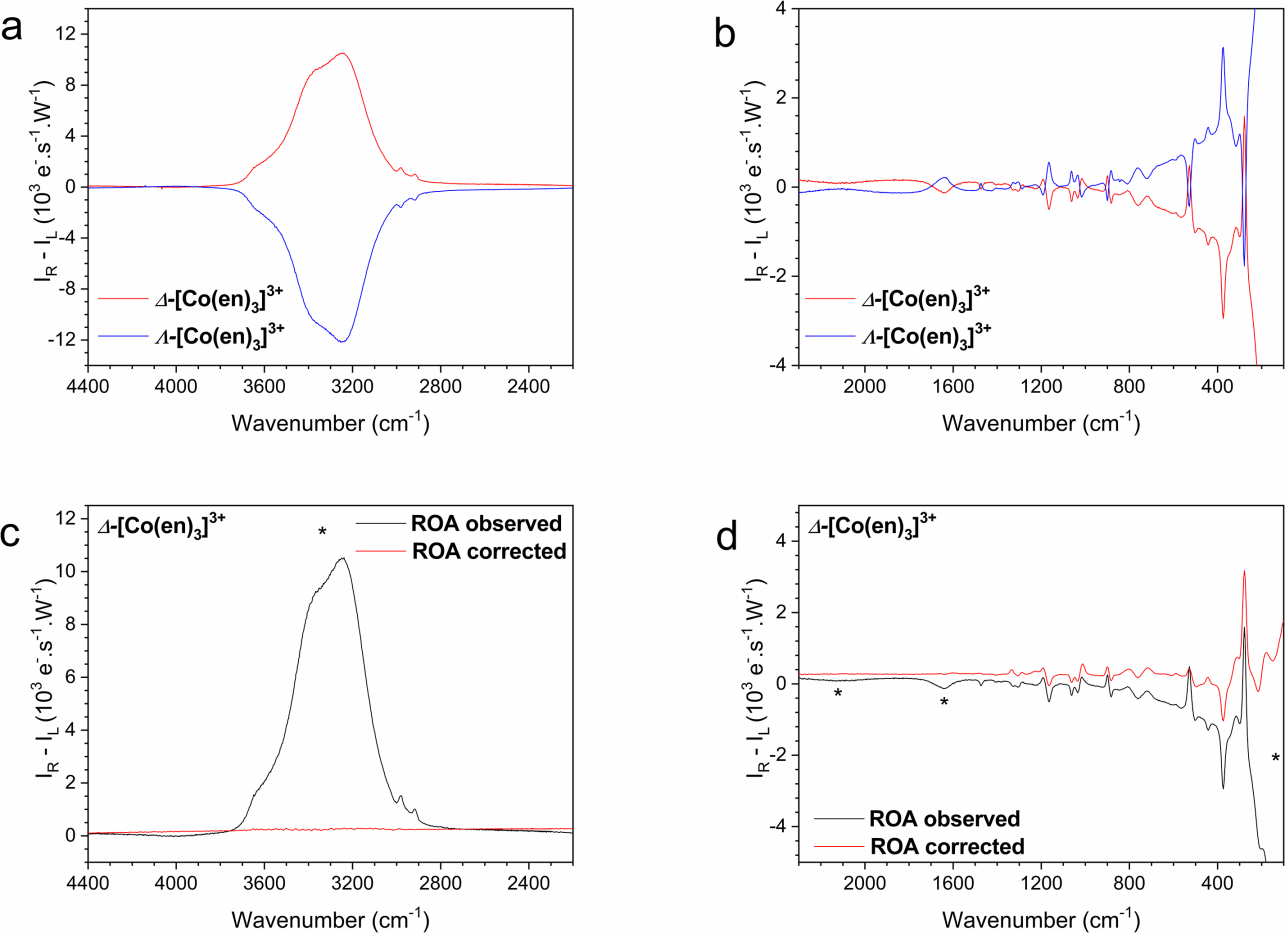
Experimental ROA spectra of ∆‐[Co(en)_3_](D(−)‐tartrate)Cl⋅5H_2_O (red) and Λ‐[Co(en)_3_](L(+)‐tartrate)Cl⋅5H_2_O (blue) collected at excitation wavelength of 532 nm with an illumination time 2 s at (a) high frequency (4400 to 2200 cm^−1^) and (b) low frequency (2300 to 100 cm^−1^). Comparison between raw ROA spectra (black) and spectra corrected for ECD‐Raman of solution (red) for Δ‐[Co(en)_3_](D(−)‐tartrate)Cl⋅5H_2_O at (c) high and (d) low frequency.

Raman and ROA spectra were recorded for both diastereomers of [Co(en)_3_](tartrate)Cl in aqueous solution (Figure 2). The Raman spectra are shown in Figure S7, and ROA spectra are presented in Figures 2a,b and S8. Taking into account the 10% difference in concentration, the ROA spectra of the Δ and Λ diastereomers exhibit a mirror–image relationship across the entire frequency range, confirming the high purity of the samples. The ROA spectra of the counter anion, sodium tartrate, were also measured in order to verify its contribution to the observed ROA spectra. Figure S9 confirms its negligible contribution, with a signal one order of magnitude lower than that of complex.

The ROA spectra display pronounced bisignate features, in agreement with earlier theoretical predictions for tris‐chelated cobalt(III) complexes under both near‐resonance and far‐from‐resonance conditions [36]. The spectral region between 100 and 2000 cm^−1^ is dominated by vibrational modes intrinsic to the [Co(en)_3_]^3+^ cation. These bands arise primarily from metal–ligand stretching, ligand deformation, and coupled skeletal vibrations of the ethylenediamine ligands.

In addition to solute‐related features, the experimental ROA spectra reveal distinct bands attributable to bulk water. These include the H‐O‐H bending mode near 1645 cm^−1^ (Figure 2d), a broad combination mode from bending and libration of water at 2100 cm^−1^, and a broad, intense feature associated with the O–H stretching modes spanning 3000–3600 cm^−1^ (Figure 2c, H_2_O modes noted with an asterisk). The presence of these solvent‐related features is unexpected based on ROA selection rules, as achiral water molecules should not directly contribute to ROA signals. Their appearance originates from the ECD–Raman contribution under near‐resonance conditions. This is confirmed by the antisymmetric chiral scattering signal in the nonzero DCP_II_ ROA spectra (Figure S10), as previously reported by Yang et al. [22].

Importantly, the overall shape of the measured ROA spectra, particularly in the low‐frequency region below 1000 cm^−1^, is also influenced by the ECD–Raman contribution from the solvent. Upon subtraction of this contribution, the intrinsic features of the pure ROA spectra of the complex are revealed. Figure 2c,d compares the raw experimental ROA spectra with the corrected spectra obtained after removal of the ECD–Raman contribution of the solution for the Δ enantiomer. The corresponding corrected spectra of the Λ enantiomer are provided in Figure S11 and comparison with the spectra of the Δ enantiomer in Figure S12.

The ECD–Raman correction procedure relies on polarization‐modulated measurements (DCP_I_ and DCP_II_) and the evaluation of CID spectra, as described in Section 2.2. These corrected experimental ROA spectra provide a robust basis for quantitative comparison with theoretical simulations, which is addressed in the following sections through DFT calculations that explicitly account for conformational averaging and solvation effects.

### 3.2 Conformational Energetics and Electronic Spectroscopy

To rationalize the experimental spectroscopic observations, DFT calculations were performed for four representative conformers of the [Co(en)_3_]^3+^ complex in aqueous solution (Figure 1): Δ(λλλ) (lel_3_), Δ(λλδ) (lel_2_ob_1_), Δ(λδδ) (lel_1_ob_2_), and Δ(δδδ) (ob_3_), following the nomenclature established in previous studies on tris‐chelated metal complexes, including related investigations on [Rh(en)_3_]^3+^ by Jorge et al. [36], Weymuth et al. [32], and Humbert‐Droz et al. [31]. A detailed description of the conformational nomenclature and computational protocol is provided in the Supporting Information. Both PCM and SMD solvent models were used to calculate the structural parameters and energy values of the Δ‐[Co(en)_3_]^3+^ conformers, with the corresponding results summarized in Tables S2 and S3.

The calculated energetic ordering of the four conformers was found to depend significantly on the chosen solvation model. Within the PCM framework, the relative stability follows the sequence:

$$\begin{array}{c} \Delta (\lambda \delta \delta )\;<\;\Delta (\delta \delta \delta )\;<\;\Delta (\lambda \lambda \lambda )\;<\;\Delta (\lambda \lambda \delta ),\\ 0.0\;(68\%)\;<\;0.14\;(18\;\%)\;<\;0.31\;(14\;\%)\;<\;2.69\;\text{kcal/mol}(<\;1\;\%), \end{array}$$

whereas the SMD model predicts a different hierarchy:

$$\begin{array}{c} \Delta (\lambda \lambda \lambda )\;<\;\Delta (\lambda \delta \delta )\;<\;\Delta (\lambda \lambda \delta )\;<\;\Delta (\delta \delta \delta ),\\ 0.0\;(17\%)\;<\;0.04\;(46\%)\;<\;0.2\;(35\%)\;<\;1.16\;\text{kcal/mol}(2\%). \end{array}$$

**FIGURE 3:**
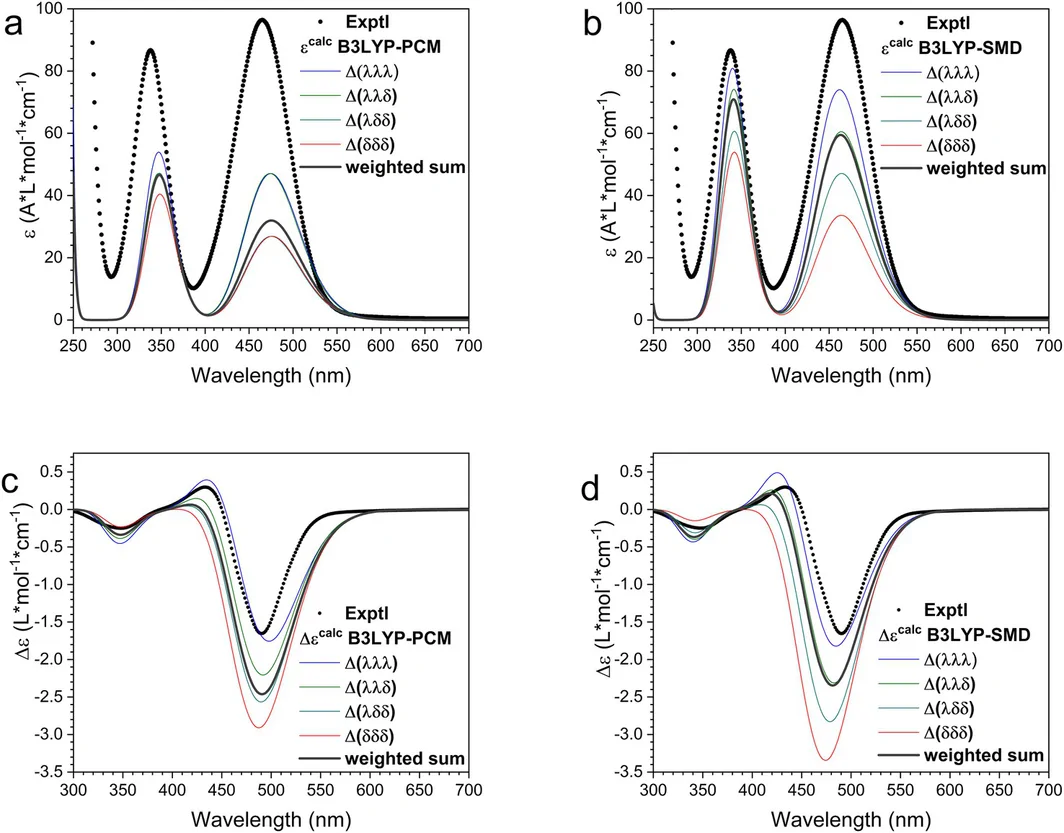
Comparison between measured and calculated UV–visible (a, b) and ECD (c, d) spectra of ∆‐[Co(en)_3_]^3+^ in water. The spectra associated with each of the possible δ and λ conformations of the en ligand (see Figure 1) calculated at the B3LYP/Def2TZVP using both PCM (a, c) and SMD (b, d) solvent models for water are displayed with their Boltzmann‐weighted sum.

To further evaluate the performance of the two solvation models, calculated UV–visible absorption and ECD spectra obtained using both PCM and SMD models were compared with the corresponding experimental spectra (Figure 3). The experimental absorption spectrum displays two prominent bands centered at approximately 465 and 340 nm (Table S1), which can be assigned to the spin‐allowed d–d transitions of the Co(III) center [35, 36], namely, the ^1^A_1_ to (^1^E, ^1^A_2_) and ^1^A_1_ to (^1^E, ^1^A_1_) transitions, respectively. At the excitation wavelength of 532 nm used for ROA measurements, the complex exhibits significant absorption and lies close to the lowest energy absorption maximum, placing the system in the near‐resonance regime. An additional intense absorption feature below 300 nm is observed but is saturated under the present experimental conditions and is attributed primarily to ligand‐centered electronic transitions.

The ECD spectrum of the Δ‐[Co(en)_3_]^3+^ enantiomer exhibits a characteristic bisignate pattern with optically active electronic transitions at approximately 490, 431.5, and 348.5 nm (Figure 3c,d), with Δε values ranging from −3.5 to +0.5 L·mol^−1^·cm^−1^. As expected, the Λ enantiomer displays mirror‐image ECD spectra, which are reported in Figure S13.

Figure 3 compares the experimentally measured absorption and ECD spectra with the calculated spectral profiles of each individual conformer, as well as with the population‐weighted sum using both PCM and SMD solvation models. For the absorption spectra, the SMD model provides a better overall agreement with the experimental data, accurately reproducing both the relative intensities and the positions of the absorption maxima. In contrast, for the ECD spectra, the PCM model yields a closer match to the experimental position of the low‐energy ECD band at λ ≈490 nm, predicting a maximum at 490.4 nm compared to 481.6 nm obtained with the SMD model.

Taken together, these results indicate that while both solvation models capture the essential features of the electronic spectra, they differ in their ability to reproduce specific experimental observables. The SMD model appears more reliable for describing absorption intensities and solvation energetics, whereas the PCM model provides a slightly improved description of the position of the lowest energy ECD transition. These differences motivate a more detailed comparison of the two models at the vibrational level, where solvent effects and conformational averaging play a critical role in determining Raman and ROA spectral signatures.

### 3.3 Vibrational Spectroscopy and ROA Simulations

Following the analysis of the electronic spectroscopic properties, a comparative investigation of the vibrational characteristics of the [Co(en)_3_]^3+^ complex was carried out using both PCM and SMD solvation models. The calculated Raman spectra for each individual conformer, as well as their population‐weighted sums, are reported in the Supporting Information. The simulated Raman profiles (Figure S1) associated with the four conformers of Δ‐[Co(en)_3_]^3+^ display largely similar spectral signatures, regardless of the solvation model employed. This indicates that Raman spectra are relatively insensitive to conformational distribution and solvent treatment, with only minor deviations observed for certain vibrational modes, particularly in the 1150–1350‐cm^−1^ region.

**FIGURE 4:**
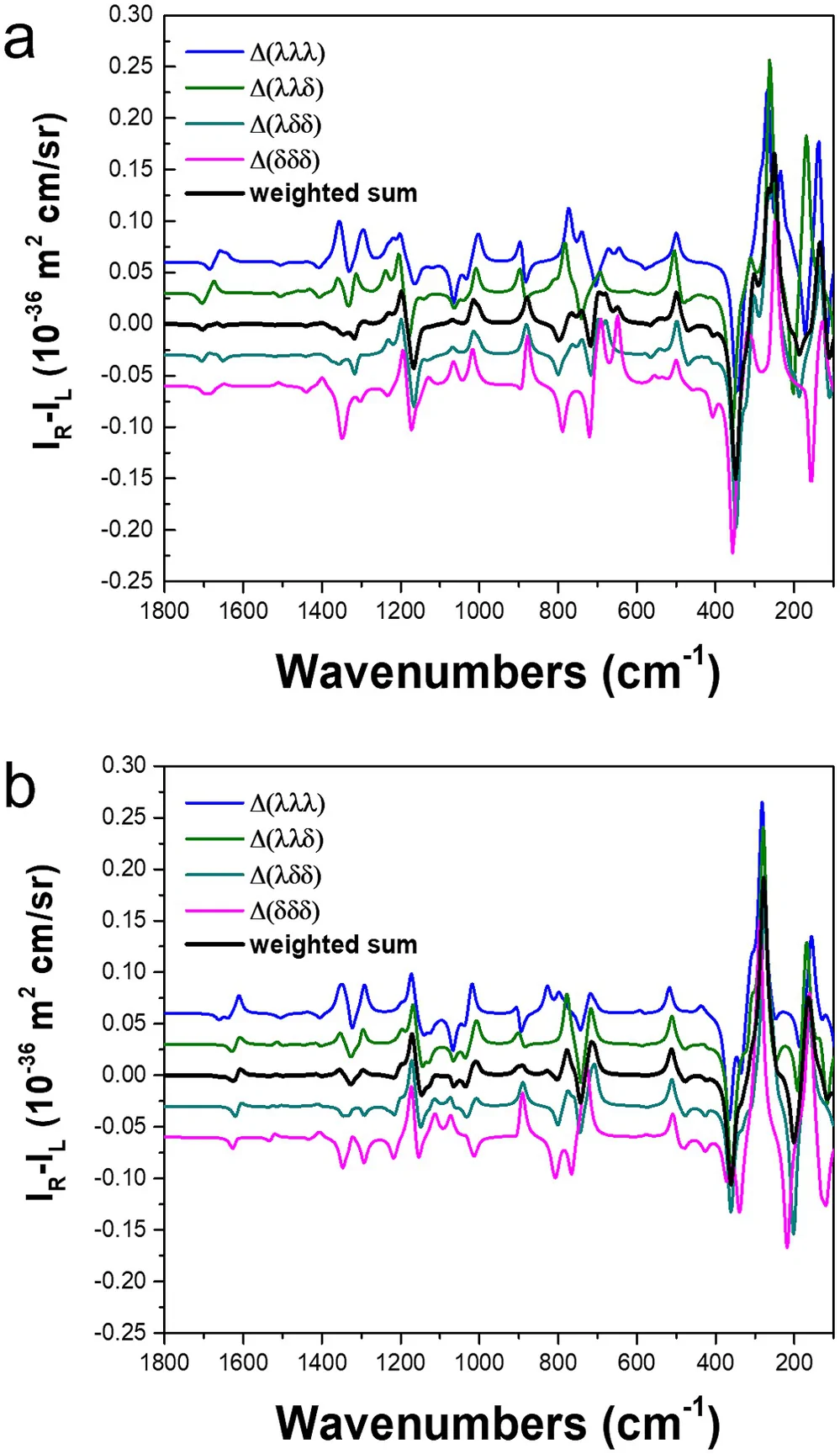
Simulated ROA for an incident wavelength of 532 nm for the four possible ∆(λλλ), ∆(λλδ), ∆(λδδ), and ∆(δδδ) conformers of ∆‐[Co(en)_3_]^3+^ in water calculated at the B3LYP/Def2TZVP level using (a) PCM and (b) SMD. For both models, the weighted sum of each of the conformational contributions is provided (black lines). For all the simulated profiles, an arbitrary FWHM of 20 cm^−1^ was applied for each calculated transition (no frequency scaling).

**FIGURE 5:**
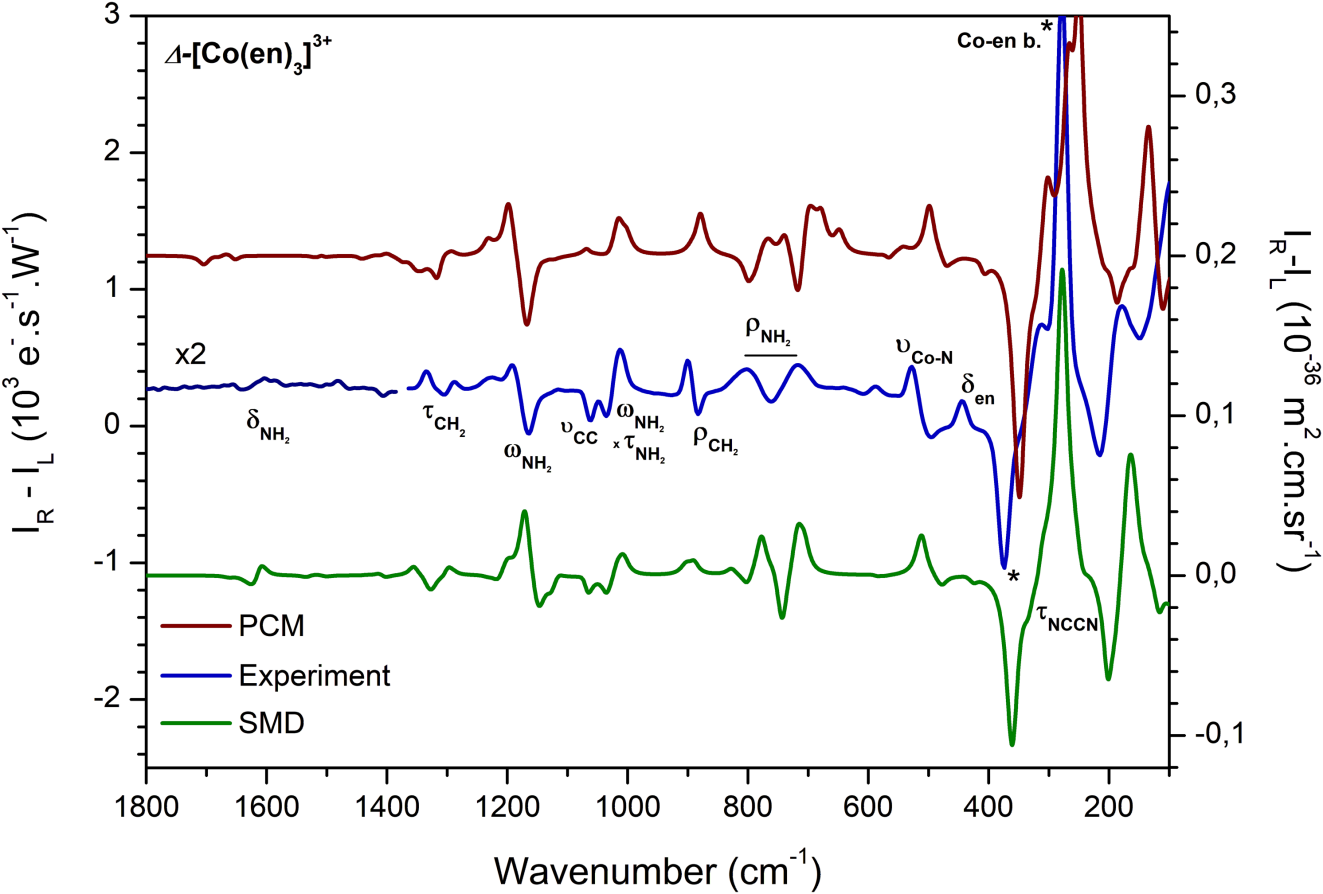
Measured (blue) and simulated ROA spectra of ∆‐[Co(en)_3_]^3+^ in water using PCM (red) and SMD (green). Spectra were corrected for ECD‐Raman (see Supporting Information). Experimental spectrum was magnified by 2 above 1350 cm^−1^ for clarity.

In contrast, the simulated ROA spectra exhibit a markedly higher sensitivity to both conformational effects and the choice of solvation model. The ROA spectra calculated at an excitation wavelength of 532 nm for each individual conformer using PCM and SMD solvation models are shown in Figure 4. A brief survey of the calculated structures shows that for a given conformer, small deviations in the structure exist between the solvent models, particularly in the metal‐ligands distances (Table S2). Accordingly, the conformers exhibit globally similar spectral signatures between models even if weak deviations in intensity and frequency can be noticed for some vibrational modes. In spite of these weak spectral changes, significant differences in both band intensities and sign patterns are observed between conformers, highlighting the importance of conformational averaging in reproducing experimental ROA spectra. Consequently, the population‐weighted ROA spectra, constructed using Boltzmann populations derived from the calculated Gibbs free energies, also display a noticeable dependence on the solvent model employed. Figure 5 compares the experimentally corrected ROA spectrum with the weighted ROA spectra obtained using PCM and SMD.

Table 1 summarizes the main experimental ROA bands and their assignments, together with the corresponding frequencies and signs obtained from DFT calculations using PCM and SMD solvation models (see also Table S3). The intense positive ROA band observed at 279 cm^−1^ is assigned to the symmetric breathing mode of the [Co(en)_3_]^3+^ core, whereas the positive feature at 314 cm^−1^ arises primarily from deformation modes of the en ligands. The negative band at 377 cm^−1^ is associated with twisting of the N–C–C–N backbone. Positive ROA bands located at 445, 496, and 528 cm^−1^ are attributed to torsion motions of the en ligands coupled with Co–N stretching vibrations. In the intermediate‐frequency region, three distinct bands observed between 716, 761, and 803 cm^−1^ are assigned predominantly to NH_2_ rocking modes. ROA features at 883, 900, 1013, and 1061 cm^−1^ correspond mainly to C–N and C–C stretching modes. At higher frequencies, bands at 1602 and 1641 cm^−1^ are assigned to NH_2_ scissoring modes. Except 1061 cm^−1^, additional features observed between 1013 and 1335 cm^−1^ arise from a coupling of CH_2_ and NH_2_ modes, as detailed in Table 1.

**TABLE 1:** Main vibrational transitions and sign of ROA intensity for Δ‐[Co(en)_3_]^3+^ conformers from the ROA spectral features (cf. Figure 5).

ν^exp^cm^−1^	*I*_ROA_	ν^theor^SMD	*I*_ROA_	ν^theor^PCM	*I*_ROA_	Assignment
178	+	164	+	135	+	C_2_‐lib_(en)_
215	−	201	−	187	−	C_2_‐lib_(en)_
279	+	278	+	266–250	+	br _Co‐(en)__3_
314	+	311	+	302	+	def_(en)_
374	−	361	−	349	−	τ_(NCCN)_
445	+	437	+			def_(en)_, δ_(en)_
496	−	477	−			ν_Co‐N_, τ^op^_(en)_
528	+	512	+	500	+	ν_Co‐N_, τ^ip^_(en)_
716	+	715	+	691	+	ρ_NH2_
761	−	743	−	720	−	ρ_NH2_
803	+	778	+	766	+	ρ_NH2_
883	−					ν_C‐N_, ρ_CH2_
900	+	891	+	879	+	ν_C‐N_, ν_C‐C_
1013	+	1009	+	1014	+	ν_C‐N_, ρ_CH2_ ⊗ τ_NH2_
1035	−	1035	−			ρ_CH2_ ⊗ τ_NH2_
1061	−	1065	−	1068	+	ν_C‐C_, ν_C‐N_
1160	−	1146	−	1168	−	ω_NH2_
1191	+	1171	+	1198	+	ω_NH__2_, τ_CH__2_
1288	+	1297	+	1293	+	τ_NH__2_, τ_CH__2_
1304	−	1327	−	1317	−	τ_CH2_
1335	+	1356	+	1348	−	ω_NH__2_, τ_CH__2_
1602 1641	+	1609 1630	+ −	1667 1704	+ −	δ_NH2_

Abbreviations: br, breathing; C_2_‐lib (en), libration‐torsion of en on C_2_ axis; def, deformation; δ, scissoring or bending; ν, stretching; ρ, rocking; τ_(ABCD)_, torsion (ip: in plane, op: out of plane); τ_AB2_, twisting; ω, wagging.

Overall, both PCM‐ and SMD‐based simulations reproduce the main experimental ROA features with good qualitative agreement. However, the SMD model provides a noticeably improved description of ROA spectral features. In particular, the NH_2_ scissoring bands at 1602 and ν = 1641 cm^−1^ are more clearly resolved in the SMD‐based calculations than in those using PCM. The characteristic doublet observed at 1160 and 1191 cm^−1^, arising from NH_2_ wagging motions, is well reproduced by both models. Furthermore, the correct signs and relative intensities of the three NH_2_ rocking bands between 716 and 803 cm^−1^ are exclusively reproduced by the SMD‐based simulations. Similarly, at low wavenumber under 530 cm^−1^, the SMD model reproduces very well the spectral features.

Taken together, these results indicate that although both solvation models yield an overall satisfactory agreement with the experimental Raman spectra, the SMD approach provides a more accurate and detailed reproduction of the ROA spectra signatures of the [Co(en)_3_]^3+^ complex in aqueous solution. This improved agreement reflects the greater reliability of the SMD model in describing solvation energetics and conformational populations.

Finally, it is instructive to compare the present results with recent theoretical work by Abella et al. [39], who reported ROA spectra for [Co(en)_3_]^3+^ considering only two conformers, Δ(δδδ) and Δ(λλλ). For these limited set of conformations, our calculated ROA profiles are in overall good agreement with their reported results, which included damping effects. Our calculations showed that inclusion of damping effects in near resonance does not significantly alter the calculated ROA spectra, which remain bisignate under both resonance and near‐resonance conditions. However, the present study clearly demonstrates that the inclusion of the intermediate hybrid conformers Δ(λδδ) and Δ(λλδ) significantly alters the energetic hierarchy and, consequently, substantially contributes to the population‐weighted ROA spectra. As a result, the incorporation of all four conformers in the calculation of the population‐weighted ROA spectra leads to a markedly improved agreement with the experimentally measured spectra.

It should be mentioned that the ROA intensities observed for [Co(en)_3_]^3+^ are significantly higher than those reported previously for [Rh(en)_3_]^3+^ under far‐from‐resonance conditions [31]. While this enhancement is likely due to the near‐resonance conditions in the [Co(en)_3_]^3+^ measurement, a decrease in polarizability with stronger ligand‐field splitting has also been proposed [39]. Therefore, the greater ROA intensity may also be attributed to the weaker ligand‐field splitting of the cobalt(III) complex compared to its rhodium(III) analogue.

## 4 Conclusion

In this work, we have investigated the electronic and vibrational chiroptical properties of Δ‐ and Λ‐[Co(en)_3_]^3+^ in aqueous solution. ROA spectra recorded under near‐resonance conditions using 532‐nm laser excitation exhibit a substantial ECD–Raman contribution arising from the solution. This contribution was quantitatively evaluated and subtracted from the measured signal, allowing reliable extraction of the intrinsic ROA spectrum of the cobalt(III) complex.

Using this experimental protocol, we show that the experimentally determined pure ROA spectra can be directly and meaningfully compared with simulated spectra obtained using first‐principles DFT. A systematic comparison between experiment and theory reveals good overall agreement, with the solvation model based on density (SMD) providing the most accurate reproduction of both the spectral features and relative intensities observed experimentally. Notably, the inclusion of four representative conformers and their Boltzmann‐weighted contributions, including degeneracy, was essential to achieving this level of agreement, underscoring the critical role of conformational averaging in ROA simulations of flexible coordination complexes.

To the best of our knowledge, this study constitutes the first experimental and theoretical investigation in which near‐resonant ROA spectra of a cationic transition‐metal complex in water are reproduced in good agreement with experiment without recourse to empirical wavenumber scaling, damping parameters, or adaptive excitation wavelength. These results highlight the robustness of the combined experimental–theoretical approach presented here and establish a general framework for future ROA investigations of chiral metal complex cations under resonant or near‐resonant conditions.

## Supporting information




**Figure S1:** Simulated SCP backscattering Raman for the four possible Δ(λλλ), Δ(λλδ), Δ(λδδ), and Δ(δδδ) conformers of complex Δ‐[Co(en)_3_]^3+^ in water calculated at the B3LYP/Def2TZVP level using PCM (upper figure) and SMD (lower figure) solvent models. For both PCM and SMD models, the weighted sum of each conformational contribution to the profile is also provided (black solid lines). A Lorentzian profile with half‐width at half‐height of 10 cm^−1^ is applied to all calculated transitions.
**Figure S2:** Simulated weighted sum of ROA spectra for the Δ‐[Co(en)_3_]^3+^ complex in water at different incident wavelengths calculated at the TD‐B3LYP/Def2TZVP level using SMD solvent model for water and compared to experimental ROA spectra of Δ‐[Co(en)_3_](D(−)‐tartrate)Cl⋅5H_2_O in water measured with an incident wavelength of 532 nm.
**Figure S3:** Simulated weighted sum of ROA spectra for Δ‐[Co(en)_3_]^3+^ complex in water at different incident wavelengths lower than 532 nm calculated at the TD‐B3LYP/Def2TZVP level using SMD solvent model for water. Note the 10^5^ factor on high intensity mono‐signate resonance ROA spectra at the incident wavelength of 465 nm.
**Figure S4:** Simulated resonant ROA spectra for Δ‐[Co(en)_3_]^3+^ calculated at incident wavelength of 465 nm for the four conformers and compared to the Boltzmann‐weighted sum total spectrum. Resonant ROA spectrum of Δ(λλλ) is dominant in intensity.
**Figure S5:** Simulated ROA spectra for Δ‐[Co(en)_3_]^3+^ calculated at 475 nm for the four conformers and compared to the Boltzmann weighted sum total spectrum. At 9 nm from the resonance, all four conformers show bisignate spectra in our model that did not take in account damping effect.
**Table S1:** First six allowed excitations for conformers of Δ‐[Co(en)_3_]^3+^ in SMD model calculation.
**Table S2:** Main structural parameters of the four conformations of the Δ‐[Co(en)_3_]^3+^ complex in water calculated at the B3LYP/def2‐TZVP level using both PCM and SMD solvent models for water (ε ≈78). Bond lengths are given in Å unit and angles in degrees. The variables d_1_ and d_2_ refer to the distances Co‐N and Co‐N′, β to the N‐Co‐N′ angle, and γ to the N‐C‐C′‐N′ dihedral angle within an ethylenediamine (en) ligand in a given conformation (δ or λ).
**Table S3:** Evaluated energies (in Hartree unit) for the four distinct conformers of the Δ‐[Co(en)_3_]^3+^ complex in water calculated at the B3LYP/def2‐TZVP level using both PCM and SMD solvent models.
**Table S4:** Evaluated energies (Gibbs energy values and energy barriers) at the Transition States associated with the three δ (γ > 0) → λ (γ < 0) conformational transformations between the distinct conformers of the Δ‐[Co(en)_3_]^3+^ complex in water (at the B3LYP/def2‐TZVP level using SMD solvent model).
**Figure S6:** Diagram scheme related to δ (γ > 0) → λ (γ < 0) conformational transformations evaluated from the calculated Δ‐[Co(en)_3_]^3+^ conformers and their transition states. Energies are indicated in kcal/mol units (cf. energy values in Table S4).
**Figure S7:** Experimental Raman spectra of 22.9‐mg/mL aqueous solution of Δ‐[Co(en)_3_](D(−)‐tartrate)Cl⋅5H_2_O at 130‐mW laser power and collection time of 39 h (red) and 19.9‐mg/mL aqueous solution of Λ‐[Co(en)_3_](L(+)‐tartrate)Cl⋅5H_2_O at 150‐mW laser power and collection time of 24 h (blue) collected at excitation wavelength of 532 nm with 2‐s illumination time.
**Figure S8:** Experimental ROA spectra of 22.9‐mg/mL aqueous solution of Δ‐[Co(en)_3_](D(−)‐tartrate)Cl⋅5H_2_O at 130‐mW laser power and collection time of 39 h (red) and 19.9‐mg/mL aqueous solution of Λ‐[Co(en)_3_](L(+)‐tartrate)Cl⋅5H_2_O at 150‐mW laser power and collection time of 24 h (blue) collected at excitation wavelength of 532 nm with 2‐s illumination time in the frequency range of detection (4400 to 100 cm^−1^). Magnified by 4 from 2300 to 100 cm^−1^.
**Figure S9:** ROA spectra for 10‐mM aqueous solution of Δ‐[Co(en)_3_](D(−)‐tartrate)Cl⋅5H_2_O (red) and Λ‐[Co(en)_3_](L(+)‐tartrate)Cl⋅5H_2_O (blue) compared with 1‐M aqueous solution of L(+)‐tartrate sodium (light green) and magnified 10 times (dark green).
**Figure S10a:** Fingerprint Raman and ROA spectra for Δ‐[Co(en)_3_](D(−)‐tartrate)Cl⋅5H_2_O and Λ‐[Co(en)_3_](L(+)‐tartrate)Cl⋅5H_2_O measured with SCP, DCP_I_, and DCP_II_ polarization modulations.
**Figure S10b:** Full range Raman and ROA spectra for Δ‐[Co(en)_3_](D(−)‐tartrate)Cl⋅5H_2_O (red line) and Λ‐[Co(en)_3_](L(+)‐tartrate)Cl⋅5H_2_O (blue line) measured with SCP, DCP_I_, and DCP_II_ polarization modulations.
**Figure S11:** Comparison between raw ROA spectra (black) and spectra corrected for ECD‐Raman of solution (blue) for 19.9‐mg/mL aqueous solution of Λ‐[Co(en)_3_](L(+)‐tartrate)Cl⋅5H_2_O collected at excitation wavelength of 532 nm with 2‐s illumination time for (a) high‐frequency range and (b) fingerprint range.
**Figure S12:** Comparison of ROA spectra corrected from ECD of Δ‐ and Λ‐ complexes collected at excitation wavelength of 532 nm. Note the perfect mirror image of corrected ROA spectra and of the symmetric background of luminescence.
**Figure S13:** Experimental ECD spectra for 56‐mM aqueous solutions of Δ‐[Co(en)_3_](D(−)‐tartrate)Cl⋅5H_2_O (red) and Λ‐[Co(en)_3_](L(+)‐tartrate)Cl⋅5H_2_O (blue) collected between 280 and 800 nm, and in the Raman range from 0 to 4500 cm^−1^.

## Data Availability

The data that support the findings of this study are available from the corresponding author upon reasonable request.
