# Supplementary material for: Near Resonant Raman Optical Activity Study of the Complex Cation Tris(ethylenediamine)cobalt(III) in Water: Experiment and DFT Calculation
Source: Chirality. 2026 Aug 27;38(9):e70128. doi: 10.1002/chir.70128 (PMC13519897; doi:10.1002/chir.70128)
Supplement: Supplementary file 1 [file CHIR-38-e70128-s001.docx]

Near Resonant Raman Optical Activity Study of the Complex Cation Tris(ethylenediamine)Cobalt(III) in water: Experiment and DFT calculation.

Maria Sara Raju^† [a]^, Nazanin Kordestani^† [b]^, Elen Duverger-Nédellec^[b]^,
Yann Danten^[a]^, Patrick Rosa^[b]^, Elizabeth A. Hillard*^[b]^, and Nicolas Daugey*^[a]^

a) *CNRS, Univ. Bordeaux, ISM, UMR 5255, 351 Cours de la Libération, 33405 Talence, France*
b) *CNRS, Univ. Bordeaux, ICMCB, UMR 5026, 87 Avenue Schweitzer, 33600 Pessac, France*

**SUPPLEMENTARY INFORMATION**

**CORRECTION OF ECD RAMAN**

The experimentally observed ROA signal can be expressed as the sum of the intrinsic ROA contribution of the solute and the ECD–Raman contribution arising from the solution^[20,22,29,30]^.

($I_{R}^{u}\text{-}I_{L}^{u}$)_observed_ = $(I_{R}^{u}\text{-}I_{L}^{u})$_pureROA_ (solute) _+_ $(I_{R}^{u}\text{-}I_{L}^{u})$_ECD-Raman_ (solution)	(1a)

In the limit of weak solute–solvent interactions, Eq. (1a) may be further approximated as:

$(I_{R}^{u}\text{-}I_{L}^{u}$)_observed_ = $(I_{R}^{u}\text{-}I_{L}^{u})$_pureROA_(solute) _+_ $(I_{R}^{u}\text{-}I_{L}^{u})$_ECD-Raman_ (solvent) _+_ $(I_{R}^{u}\text{-}I_{L}^{u})$_ECD-Raman_ (solute)	(1b)

The ECD-Raman contribution $(I_{R}^{u}\text{-}I_{L}^{u})$_ECD-Raman_ was calculated and subtracted from the raw $\left( I_{R}^{u}\text{-}I_{L}^{u} \right)$_observed_ spectra according to the following equations:

${\text{(}\text{I}_{\text{R}}^{\text{u}}\text{-}\text{I}_{\text{L}}^{\text{u}}\text{)}}_{ECD\text{-}Ram}\text{= }{CID}_{ECD\text{-}Raman}\text{ }.\text{ }\text{(I}_{\text{R}}^{\text{u}}\text{+}\text{I}_{\text{L}}^{\text{u}})$	(2a)

where the Circular Intensity Difference (CID) associated with the ECD–Raman effect is defined as:

${CID}_{ECD\text{-}Ram}\text{= }\frac{I_{R}\text{-}I_{L}\text{ }}{I_{R}\text{+}I_{L}}\text{ }\cong f\text{ }\frac{\Delta\varepsilon^{'}\text{+}DoC\text{ }.\text{ }\Delta\varepsilon}{4}cLln(10)$	(2b)

In Eq. (2b), Δε and Δε′ represent the decadic molar absorption coefficient differences at the incident and scattered wavelengths, respectively (Figure S13); *c* is the solution concentration; and *L* is the effective scattering path length. The factor *f* is given by:

$f=1+\frac{2L^{'}}{L}$

Where, *L*′ is the optical path length over which absorption occurs without Raman scattering.

The Degree of Circularity spectra (DoC) is calculated from the DCP measurements according to:

$DoC\text{=}\frac{({DCP}_{I}\text{-}{DCP}_{II})}{({DCP}_{I}\text{+}{DCP}_{II})}$	(2c)

Here, DCP_I_ and DCP_II_ correspond to dual circular polarization scattering signals involving the combinations $(I_{R}^{R}\text{±}I_{L}^{L}$) and $(I_{L}^{R}\text{±}I_{R}^{L}$), respectively (Figure S10a-b). The resulting *CID_ECD-Raman_* spectrum is multiplied by the total Raman intensity to obtain the ECD-Raman contribution to the detected $I_{R}\text{-}I_{L}$ signal. This contribution is then subtracted from the observed spectrum to yield the pure ROA signal of the complex^[20,22,29,30]^.

**COMPUTATIONAL DETAILS**

**Raman and ROA spectra for stable conformers of Δ-[Co(en)3]^3+^**

We display in Figure S1 the calculated (backscattering) Raman spectra (at incident wavelength of 532 nm) associated with each distinct conformers of complex Δ-[Co(en)_3_]^3+^ in water using PCM or SMD solvent model.

**Figure S1:** Simulated SCP backscattering Raman for the four possible Δ(λλλ), Δ(λλδ), Δ(λδδ) and Δ(δδδ) conformers of complex Δ-[Co(en)_3_]^3+^ in water calculated at the B3LYP/Def2TZVP level using PCM (upper figure) and SMD (lower figure) solvent models. For both PCM and SMD models the weighted sum of each conformational contribution to the profile is also provided (black solid lines). A Lorentzian profile with half-width at half-height of 10 cm^-1^ is applied to all calculated transitions.

Are also reported in this figure, the weighted sum of each conformational contribution to the spectra obtained from the distribution of the population of these conformers as evaluated using PCM or SMD solvent model (Table S3). The simulated Raman profiles associated with each possible conformers of Δ-[Co(en)_3_]^3+^ exhibit more or less the same main spectral signatures and this independently of the solvent model used (PCM versus SMD), even if slight deviations are perceptible for some specific modes especially in the spectral range 1150-1350 cm^-1^. In contrast, the simulated ROA profiles associated with each conformer of Δ-[Co(en)_3_]^3+^ are globally much more sensitive to the solvent model used. According to the solvent model used for one conformer, the positions of the calculated vibrational transitions does not differ considerably, whereas the ROA activity and in particular their sign can be significantly affected. However, the main difference comes out from the Boltzmann population of the distinct conformers which varies from the solvent model used, PCM favoring oblique conformations of (en) ligand whereas SMD favoring quite parallel ones.

**Resonance effect on ROA spectra calculated with TD-B3LYP/Def2TZVP level using SMD solvent model for water without damping:**

In Figure S2, simulated ROA spectra for Δ-[Co(en)_3_]^3+^ complex at excitation wavelength from 1319 (λ_i_) to 532 (λ_f_) nm exhibit a greater increase than theoretically expected by scattering factor (λ_i_/λ_f_)^5^. Especially between 1064 and 532 nm, the ratio of ROA intensity is estimated of more than 100, much higher than the theoretical scattering value of (1064/532)^5^ = 32, confirming near resonance at 532nm.

Figure S3 provides a comparison between simulated ROA spectra at excitation wavelength from 532 nm to 465 nm under resonance exhibits a very high ROA intensity ratio about 10^5^ without damping effect. Moreover, at the resonance at 465 nm the ROA spectrum is monosignated as well as at 470 nm. Figure S4 shows the ROA monosignated spectra of the four conformers at 465 nm (i.e. very close of the calculated electronic absorption transition in bold Table S1 (< 1 nm, cf. Figure 3 of the simulated/measured UV and ECD spectra). Thus, even if the Δ(λλλ) (lel3) population to weighted sum ROA spectra is low (p_lel3_ = 0.165), its contribution on the ROA spectra at the resonance predominates with an intensity more than 200 times higher than other conformers. At 475 nm (Figure S5), all the simulated ROA spectra are bisignated. Notice that the observed ECD maximum around 485 nm is well-reproduced by DFT-calculations and results from the weighted sum of the ECD transitions of conformers located at 460-467 nm. Finally, the nature bisignated or monosignated of the simulated spectra strongly depends on the proximity of the excitation wavelength to the electronic transitions associated to each conformer in solution as well as the shape of the final spectrum.

**Figure S2:** Simulated weighted sum of ROA spectra for the Δ-[Co(en)_3_]^3+^ complex in water at different incident wavelengths calculated at the TD-B3LYP/Def2TZVP level using SMD solvent model for water and compared to experimental ROA spectra of Δ-[Co(en)_3_](D(−)-tartrate)Cl⋅5H_2_O in water measured with an incident wavelength of 532 nm.

**Figure S3:** Simulated weighted sum of ROA spectra for Δ-[Co(en)_3_]^3+^ complex in water at different incident wavelengths lower than 532 nm calculated at the TD-B3LYP/Def2TZVP level using SMD solvent model for water. Note the 10^5^ factor on high intensity monosignate resonance ROA spectra at the incident wavelength of 465 nm.

**Figure S4:** Simulated resonant ROA spectra for Δ-[Co(en)_3_]^3+^ calculated at incident wavelength of 465 nm for the four conformers and compared to the Boltzmann weighted sum total spectrum. Resonant ROA spectrum of Δ(λλλ) is dominant in intensity.

**Figure S5:** Simulated ROA spectra for Δ-[Co(en)_3_]^3+^ calculated at 475 nm for the four conformers and compared to the Boltzmann weigthed sum total spectrum. At 9 nm from the resonance, all four conformers show bisignate spectra in our model that did not take in account damping effect.

**Table S1:** First six allowed excitations for conformers of Δ-[Co(en)_3_]^3+^ in SMD model calculation.

| - **(nm)** | **F** | **ε (L mol^-1^ cm^-1^)** | **Δε (L mol^-1^ cm^-1^)** | **R (10^-40^esu^2^ cm^2^)** |
| --- | --- | --- | --- | --- |
| **Δ(λλλ) lel3** |  |  |  |  |
| **464.50** | **0.0002** | **839.77** | **-24.13** | **-410.19** |
| 464.17 | 0.0002 | 839.77 | -23.94 | -407.36 |
| 460.30 | 0.0007 | 2939.19 | 43.60 | 747.98 |
| 340.06 | 0.0006 | 2519.31 | -0.56 | -13.11 |
| 340.03 | 0.0006 | 2519.31 | -0.58 | -13.42 |
| 339.26 | 0 | 0.00 | -0.02 | -0.43 |
| **Δ(λλδ) lel2-ob1** |  |  |  |  |
| 466.46 | 0.0002 | 847.89 | -22.20 | -379.41 |
| **465.92** | **0.0002** | **847.89** | **-22.30** | **-381.55** |
| 461.76 | 0.0005 | 2119.73 | 37.65 | 650.10 |
| 342.09 | 0.0006 | 2543.68 | -0.84 | -19.51 |
| 340.89 | 0.0005 | 2119.73 | -0.22 | -5.23 |
| 338.88 | 0 | 0.00 | -0.01 | -0.32 |
| **Δ(λδδ) lel1-ob2** |  |  |  |  |
| 467.29 | 0.0001 | 424.94 | -20.27 | -346.69 |
| **465.90** | **0.0002** | **849.88** | **-22.75** | **-390.23** |
| 462.41 | 0.0004 | 1699.75 | 33.60 | 580.66 |
| 342.44 | 0.0005 | 2124.69 | -0.73 | -17.14 |
| 341.6 | 0.0004 | 1699.75 | -0.07 | -1.70 |
| 338.42 | 0 | 0.00 | -0.03 | -0.78 |
| **Δ(δδδ) ob3** |  |  |  |  |
| 466.32 | 0.0001 | 422.19 | -20.25 | -344.84 |
| **465.85** | **0.0001** | **422.19** | **-19.74** | **-336.46** |
| 462.40 | 0.0003 | 1266.58 | 28.17 | 483.80 |
| 342.16 | 0.0004 | 1688.78 | -0.24 | -5.57 |
| 341.96 | 0.0004 | 1688.78 | -0.16 | -3.75 |
| 337.71 | 0 | 0.00 | 0.00 | -0.09 |

ε molar extinction, Δε differential molar extinction, f oscillator strengths, R Rotatory strengths in 10^−40^ cgs

**Table S2:** Main structural parameters of the four conformations of the Δ-[Co(en)_3_]^3+^ complex in water calculated at the B3LYP/def2-TZVP level using both PCM and SMD solvent models for water (ε≈78). Bond lengths are given in Å unit and angles in degrees. The variables d_1_ and d_2_ refer to the distances Co-N and Co-N’, β to the N-Co-N’ angle and γ to the N-C-C’-N’ dihedral angle within an ethylenediamine (en) ligand in a given conformation (δ or λ).

|  |  | **en_1_** | **en_2_** | **en_3_** |
| --- | --- | --- | --- | --- |
| **PCM (water)** |  | **λ** | **λ** | **λ** |
| **Δ(λλλ)** | **d_1_ (Å)** | 2.0053 | 2.0059 | 2.0047 |
| **(or lel3)** | **d_2_ (Å)** | 2.0080 | 2.0045 | 2.0065 |
|  | $\bar{\mathbf{d}}$ **(Å)** | 2.0058±0.0013 | | |
|  | **β** | 84.3 | 84.5 | 84.4 |
|  | **γ** | -48.9 | -49.4 | -49.2 |
| **Δ(λλδ)** |  | **δ** | **λ** | **λ** |
| **(or lel2-ob1)** | **d_1_ (Å)** | 2.0038 | 2.0064 | 2.0064 |
|  | **d_2_ (Å)** | 2.0038 | 2.0035 | 2.0035 |
|  | $\bar{\mathbf{d}}$ **(Å)** | 2.0046±0.0014 | | |
|  | **β** | 84.3 | 84.4 | 84.4 |
|  | **γ** | 48.3 | -49.9 | -49.9 |
| **Δ(λδδ)** |  | **δ** | **δ** | **λ** |
| **(or lel1-ob2)** | **d_1_ (Å)** | 2.0056 | 2.0063 | 2.0003 |
|  | **d_2_ (Å)** | 2.0068 | 2.0064 | 2.0084 |
|  | $\bar{\mathbf{d}}$ **(Å)** | 2.0056±0.0028 | | |
|  | **β** | 84.0 | 84.0 | 84.1 |
|  | **γ** | 48.6 | 49.0 | -47.4 |
| **Δ(δδδ)** |  | **δ** | **δ** | **δ** |
| **(or ob3)** | **d_1_ (Å)** | 2.0057 | 2.0071 | 2.0068 |
|  | **d_2_ (Å)** | 2.0057 | 2.0059 | 2.0070 |
|  | $\bar{\mathbf{d}}$ **(Å)** | 2.0064±0.0007 | | |
|  | **β** | 84.0 | 84,0 | 84.0 |
|  | **γ** | 48.1 | 47.7 | 47.9 |
| **SMD (water)** |  | **λ** | **λ** | **λ** |
| **Δ(λλλ)** | **d_1_ (Å)** | 1.9912 | 1.9936 | 1.9906 |
| **(or lel3)** | **d_2_ (Å)** | 1.9902 | 1.9930 | 1.9933 |
|  | $\bar{\mathbf{d}}$ **(Å)** | 1.9920±0.0015 | | |
|  | **β** | 84.9 | 84.8 | 84.9 |
|  | **γ** | -50.4 | -50.8 | -50.4 |
| **Δ(λλδ)** |  | **δ** | **λ** | **λ** |
| **(or lel2-ob1)** | **d_1_ (Å)** | 1.9937 | 1.9920 | 1.9940 |
|  | **d_2_ (Å)** | 1.9944 | 1.9918 | 1.9945 |
|  | $\bar{\mathbf{d}}$ **(Å)** | 1.9934±0.0012 | | |
|  | **β** | 84.5 | 84.6 | 84.6 |
|  | **γ** | 49.2 | -49.5 | -49.7 |
| **Δ(λδδ)** |  | **δ** | **δ** | **λ** |
| **(or lel1-ob2)** | **d_1_ (Å)** | 1.9925 | 1.9955 | 1.9924 |
|  | **d_2_ (Å)** | 1.9963 | 1.9946 | 1.9938 |
|  | $\bar{\mathbf{d}}$ **(Å)** | 1.9942±0.0016 | | |
|  | **β** | 84.4 | 84.3 | 84.4 |
|  | **γ** | 49.0 | 49.0 | -49.3 |
| **Δ(δδδ)** |  | **δ** | **δ** | **δ** |
| **(or ob3)** | **d_1_ (Å)** | 1.9947 | 1.9929 | 1.9946 |
|  | **d_2_ (Å)** | 1.9942 | 1.9951 | 1.9943 |
|  | $\bar{\mathbf{d}}$ **(Å)** | 1.9943±0.0008 | | |
|  | **β** | 84.3 | 84.3 | 84.3 |
|  | **γ** | 48.2 | 49.5 | 48.6 |

ǂ $\bar{\mathbf{d}}$ **(Å)** is the mean value (± the rms value) of the six Co-N (metal-ligand) distances for the calculated Δ-[Co(en)_3_]^3+^ complex in a given conformation.

**Table S3:** Evaluated energies (in Hartree unit) for the four distinct conformers of the Δ-[Co(en)_3_]^3+^ complex in water calculated at the B3LYP/def2-TZVP level using both PCM and SMD solvent models.

|  | **Δ-[Co(en)3]^3+^ conformations** | | | |
| --- | --- | --- | --- | --- |
| **PCM (water)** | **Δ(δδδ)**  **(or ob3)** | **Δ(λδδ)**  **(or lel1-ob2)** | **Δ(λλδ)**  **(or lel2-ob1)** | **Δ(λλλ)**  **(or lel3)** |
| **E°** (H) | -1954.327679 | -1954.327252 | -1954.32715 | -1954.32599 |
| **E°**^+ZPE^ (H) | -1953.971474 | -1953.971595 | -1953.969213 | -1953.97146 |
| **U^298K^** (H) | -1953.956789 | 1953.956792 | -1953.955169 | -1953.9571 |
| **H^298K^**(H) | -1953.955845 | -1953.955848 | -1953.954224 | -1953.95616 |
| **G^298K^** (H) | -1954.011877 | **-1954.012098** | -1954.007805 | -1954.01161 |
| **δG^298K^** (kcal/mol) | 0.139 | 0.0 | 2.69 | 0.31 |
| **wg(Δ(ijk))** | 0.179 | 0.679 | 0.007 | 0.135 |
| **SMD (water)** | **Δ(δδδ)**  **(or ob3)** | **Δ(λδδ)**  **(or lel1-ob2)** | **Δ(λλδ)**  **(or lel2-ob1)** | **Δ(λλλ)**  **(or lel3)** |
| **E°** (H) | -1954.387532 | -1954.387367 | -1954.387605 | -1954.387865 |
| **E°**^+ZPE^ (H) | -1954.030735 | -1954.031719 | -1954.031914 | -1954.031973 |
| **U^298K^** (H) | -1954.01695 | -1954.017527 | -1954.01785 | -1954.017918 |
| **H^298K^**(H) | -1954.016006 | -1954.016583 | -1954.0169 | -1954.016974 |
| **G^298K^** (H) | -1954.068878 | -1954.070660 | -1954.07041 | **-1954.070729** |
| **δG^298K^** (kcal/mol) | 1.16 | 0.04 | 0.20 | 0.0 |
| **wg(Δ(ijk))** | 0.023 | 0.459 | 0.353 | 0.165 |

Population of each possible conformer in solution is evaluated from the Maxwell-Boltzmann distribution (p_i_ = g_i_.exp(-ΔE_i_/k_B_T) / ∑_i_ g_i_.exp(-ΔE_i_/k_B_T) and so ∑_i_ p_i_ = 1) in which intermediate forms λδδ and λλδ of tris-chelated distinct (en) ligands assuming either λ- or δ-conformation are statistically favored (g_i_=3) over δδδ and λλλ forms.

**Transition States for δ- (γ>0) → λ- (γ<0) transformations between Δ-[Co(en)_3_]^3+^ conformers**

We have also evaluated PES surfaces (not shown and only calculated at the B3LYP/Def2TZVP level using SMD water model) to determine transition states and energy barriers associated with the three δ- (γ>0) → λ- (γ<0) conformational transformations 1) ob1-lel2 ← lel3 2) ob2-lel1 ← ob1-lel2 and 3) ob3 ← ob2-lel1. Such a transformation is carried out by varing the the dihedral angle γ of a diamine-ethylene ligand from δ- (γ>0) to λ- (γ<0) conformation, here (cf. Figure S6 for diagram scheme).

**Table S4:** Evaluated energies (Gibbs energy values and energy barriers) at the Transition States associated with the three δ- (γ>0) → λ- (γ<0) conformational transformations between the distinct conformers of the Δ-[Co(en)_3_]^3+^ complex in water (at the B3LYP/def2-TZVP level using SMD solvent model).

|  | **TS1** | **TS2** | **TS3** |
| --- | --- | --- | --- |
|  | **ob1-lel2 ← lel3** | **ob2-lel1 ← ob1-lel2** | **ob3 ← ob2-lel1** |
| **G^298K^(H)** | -1954.060715 | -1954.062744 | -1954.061760 |
| **ΔGǂ (kcal/mol)** | +6.2 | +5.0 | +5.6 |

In this table, ΔGǂ (kcal/mol) is the energy barrier associated with δ- (γ>0) → λ- (γ<0) transformation evaluated as the difference between the Gibbs energy of the transition state and the Gibbs energy of the more stable conformer (as a reference) associated with the ob2-lel1 conformer using the SMD water model.

**Figure S6:** Diagram Scheme related to δ (γ>0) → λ (γ<0) conformational transformations evaluated from the calculated Δ-[Co(en)_3_]^3+^ conformers and their transition states. Energies are indicated in kcal/mol units (cf. energy values in Table S4).

The main steps of the protocol used to localize the transition states are the following. In a first step, we evaluated numerically at the B3LYP/Def2TZVP level of calculation (using the SMD solvent model) the Potential Energy Surface (PES) associated with a given transformation from one conformer to another. This was done by varying the dihedral γ-value (with a step of 2.5°) of one of the diamine ethylene (en) ligands within the Δ-[Co(en)₃]³^+^ complex from the λ-conformation (λ<0 for the Δ-enantiomer) to the δ-conformation (λ>0 for the Δ-enantiomer). This leads to the evaluation of three independent PES for the transformations Δ(λλλ)→Δ(λλδ), Δ(λλδ)→Δ(λδδ) and Δ(λδδ)→Δ(δδδ). A preliminary examination of the PES allows a rough localization of the transition states (TS1, TS2 and TS3, respectively), which are associated with both conformers involved in the transformation. The localization of each transition state was then refined and the optimization of both minima and transition state (all the coordinates are optimized) was completed using a *Synchronous Transit-Guided Quasi-Newton* (STQN) method. ^[1]^ After locating these stationary points (TS and both minima), the linking of the transition state with both adjacent minima was ensured by explicitly computing the reaction path using the intrinsic reaction coordinate (IRC) integration method.^[2]^ Finally, according to this procedure (PES analysis and IRC calculations), the nature of the obtained transition state was fully characterized by performing a normal mode vibrational analysis. This allows the elaboration of the energy diagram associated with the three conformational transformations as displayed in Figure S6.

**EXPERIMENTAL SECTION**

**Figure S7:** Experimental Raman spectra of 22.9 mg/mL aqueous solution of Δ-[Co(en)_3_](D(−)-tartrate)Cl⋅5H_2_O at 130 mW laser power and collection time of 39 hours (red) and 19.9 mg/mL aqueous solution of Λ-[Co(en)_3_](L(+)-tartrate)Cl⋅5H_2_O at 150 mW laser power and collection time of 24 hours (blue) collected at excitation wavelength of 532 nm with 2 s illumination time.

**Figure S8:** Experimental ROA spectra of 22.9 mg/mL aqueous solution of Δ-[Co(en)_3_](D(−)-tartrate)Cl⋅5H_2_O at 130 mW laser power and collection time of 39 hours (red) and 19.9 mg/mL aqueous solution of Λ-[Co(en)_3_](L(+)-tartrate)Cl⋅5H_2_O at 150 mW laser power and collection time of 24 hours (blue) collected at excitation wavelength of 532 nm with 2 s illumination time in the frequency range of detection (4400 cm^-1^ to 100 cm^-1^). Magnified by 4 from 2300 cm^-1^ to 100 cm^-1^.

**Figure S9:** ROA spectra for 10 mM aqueous solution of Δ-[Co(en)_3_](D(−)-tartrate)Cl⋅5H_2_O (red) and Λ-[Co(en)_3_](L(+)-tartrate)Cl⋅5H_2_O (blue) compared with 1 M aqueous solution of L(+)-tartrate sodium (light green) and magnified 10 times (dark green).

 **Figure S10a:** Fingerprint Raman and ROA spectra for Δ-[Co(en)_3_](D(−)-tartrate)Cl⋅5H_2_O and Λ- [Co(en)_3_](L(+)-tartrate)Cl⋅5H_2_O measured with SCP, DCP_I_ and DCP_II_ polarization modulations.

 **Figure S10b:** Full range Raman and ROA spectra for Δ-[Co(en)_3_](D(−)-tartrate)Cl⋅5H_2_O (red line) and Λ-[Co(en)_3_](L(+)-tartrate)Cl⋅5H_2_O (blue line) measured with SCP, DCP_I_ and DCP_II_ polarization modulations.

**Figure S11:** Comparison between raw ROA spectra (black) and spectra corrected for ECD-Raman of solution (blue) for 19.9 mg/mL aqueous solution of Λ-[Co(en)_3_](L(+)-tartrate)Cl⋅5H_2_O collected at excitation wavelength of 532 nm with 2s illumination time for (a) high frequency range and (b) fingerprint range.

**Figure S12:** Comparison of ROA spectra corrected from ECD of Δ- and Λ- complexes collected at excitation wavelength of 532 nm. Note the perfect mirror image of corrected ROA spectra and of the symmetric background of luminescence.

**Figure S13:** Experimental ECD spectra for 56 mM aqueous solutions of Δ-[Co(en)_3_](D(−)-tartrate)Cl⋅5H_2_O (red) and Λ-[Co(en)_3_](L(+)-tartrate)Cl⋅5H_2_O (blue) collected between 280 – 800 nm, and in the Raman range from 0 to 4500 cm^-1^.

1. Ayala, P. Y.; Schlegel, H. B. A Combined Method for Determining Reation Paths, Minima and Transition State Geometries J. Chem. Phys. **1997**, 107, 375– 384. DOI: 10.1063/1.474398.

   Peng, C.; Ayala, P. Y.; Schlegel, H. B.; Frisch, M. J. Using Redundant Internal Coordinates to Optimize Equilibrium Geometries and Transition States J. Comput. Chem. **1996**, 17, 49– 56. DOI: 10.1002/(SICI)1096-987X(19960115)17:1<49::AID-JCC5>3.0.CO;2-0.

   Peng, C.; Schlegel, H. B. Combining Synchronous Transit and Quasi-Newton Methods for Finding Transition States Isr. J. Chem. **1993**, 33, 449– 54 DOI: 10.1002/ijch.199300051.
2. Hratchian, H. P.; Schlegel, H. B. Using Hessian Updating to Increase the Efficiency of a Hessian Based Predictor-corrector Reaction Path Following Method J. Chem. Theory Comput. **2005**, 1, 61– 69 DOI: 10.1021/ct0499783
